# Cross-cultural adaptation and validation of the CFAbd-Score for gastrointestinal symptoms in patients with cystic fibrosis

**DOI:** 10.1016/j.jped.2024.07.004

**Published:** 2024-08-10

**Authors:** Flávia N.S. Infante, Elizete A. Lomazi, Carlos Zagoya, Franziska Duckstein, Daniela O. Magro, Fernando Pessotto, Antônio F. Ribeiro, José D. Ribeiro, Jochen G. Mainz

**Affiliations:** a Universidade Estadual de Campinas (UNICAMP), Pós-Graduação em Saúde da Criança e do Adolescente, Campinas, SP, Brazil; b Centro Universitário UniAnchieta, Faculdade de Nutrição, Jundiaí, SP, Brazil; c Universidade Estadual de Campinas (UNICAMP), Faculdade de Ciências Médicas (FCM), Departamento de Pediatria, Campinas, SP, Brazil; d University, Klinikum Westbrandenburg, Cystic Fibrosis Centre, Brandenburg Medical School (MHB), Brandenburg an der Havel, Germany; e Universidade Estadual de Campinas (UNICAMP), Faculdade de Ciências Médicas (FCM), Departamento de Cirurgia, Campinas, SP, Brazil; f Centro Universitário UniAnchieta, Faculdade de Psicologia, Jundiaí, SP, Brazil

**Keywords:** Cystic fibrosis, Validation study, Gastrointestinal tract, Quality of life, Patient-reported outcome measures, Questionnaire

## Abstract

**Objective:**

Translating and cross-culturally adapting the CFAbd-Score, Cystic Fibrosis (CF) Abdominal Score, to use in Brazilian spoken Portuguese. The CFAbd-Score is a questionnaire for assessing CF-related abdominal symptoms and their influence on the quality of life (QoL). It comprises 28 questions on five domains: abdominal pain, bowel movements, eating and appetite, gastroesophageal reflux symptoms, and the impact of gastrointestinal (GI) symptoms on QoL.

**Method:**

Cross-cultural adaptation included assessment of conceptual and item equivalence, semantic, operational, and measurement equivalence. Content validity was assessed. The validation and psychometric analysis phase included 97 people with CF (pwCF), median age:14.58y (IQR 9/19), and 105 healthy individuals, 15.10y (IQR 9/20). Exploratory factor analysis (FA) identified retained factors. Internal consistency of the extracted domains was evaluated using Cronbach's α, and the Kaiser-Meyer-Olkin test (KMO) was used to check the sample adequacy. Bartlett's test tested the null hypothesis that the correlation matrix is an identity matrix.

**Results:**

All items were considered relevant to the construct and good semantic equivalence of the version was recognized. FA showed the appropriate weight of all items and good internal consistency, with Cronbach's alpha 0.89. Bartlett's test significance level (p < 0.001) and KMO coefficient of 0.72 indicated good adequacy for structure. Internal consistency coefficients (Cronbach's alpha) were good for abdominal pain: 0.84; abdominal bloating: 0.73; flatulence: 0.76; heartburn: 0.81, and low for reflux: 0.54.

**Conclusion:**

The CFAbd-Score was adapted to the Brazilian spoken Portuguese and demonstrated content and semantic equivalence. The final version showed appropriate validity, and internal consistency, preserving the psychometric properties of the original version.

## Introduction

Cystic fibrosis (CF) is the most common genetic disorder in Caucasian individuals, with an incidence of 1/2,500–3,500 live births. It is an autosomal recessive genetic disorder caused by mutations in (*CFTR*)-encoding gene, the cystic fibrosis transmembrane conductance regulator (CFTR) gene. The CFTR protein is expressed in multiple organs and determines systemic involvement. Clinical manifestations occur early in life involving the airways and the gastrointestinal tract (GI) as crucial organs.^1^

Nowadays, 6427 Brazilian individuals are registered in the CF Brazilian Registry database.^2^ Around 75% of these are up to 18 years old, the proportion of adults is still less than 30% of the total and only 7.7% are over the age of 30. In Brazil, the CF population is predominantly pediatric while in developed countries, the adult population already exceeds that of children and adolescents.

GI symptoms are common in pwCF, in an active surveillance study published in 2017, all patients reported at least one abdominal symptom during the previous three months.^3^ The high frequency of abdominal pain and other GI symptoms prompted the Cystic Fibrosis Foundation to develop a medical training program for the care and research of GI manifestations in pwCF.^4^ To meet this need, German researchers conceived and validated the first CF-specific questionnaire (CFAbd-Score) following United States Food and Drug Administration guidelines for the development of a patient-reported outcome measure (PROM) for systematically assessing GI symptoms in pwCF.^5^

A patient-centered instrument to measure these symptoms will be very useful given the recent introduction of new modulators of the CFTR delivered by Brazilian government.^6^

The development of a questionnaire is time-consuming and requires personnel and financial investment, so, translation, cross-cultural adaptation, and validation of original questionnaires are considered an advantaged option.^7^ This study aimed to cross-culturally adapt and validate the German CFAbd-Score for clinical and research objectives targeting Brazilian pwCF.

## Methods

### Type of study

A validation study, translation, and cross-cultural adaptation study.

### Population

PwCF from the age of six, attending the outpatient clinic at a Brazilian university CF-Reference Center. A group of healthy individuals was recruited from a private high school.

### Inclusion criteria

CF-patients seen at the outpatient clinic of the University CF Reference Center, with diagnosis defined by genetic and/or sweat testing, aged six or over when entered the study. Consent from legal guardians and/or parents and participants by signing the Informed Consent Form (ICF) and the consent forms for participants under 18 years old.

For healthy individuals, the inclusion criteria are age under 6, being not diagnosed with any disease, having consented by signing the ICF, and assenting (for those under 18) by signing the consent form.

### Exclusion criteria

Patients and/or guardians who do not sign the ICF, those who do not complete the questionnaire, patients who do not have a definitive CF diagnosis, and patients under six years old.

For healthy individuals, the exclusion criteria are being under six years old, having a diagnosis of any disease, reported abdominal problems owing to food intolerances, allergies, and/or celiac diseases, not having consented by signing the ICF or assenting (for those under 18y), and not answering all the items in the questionnaire.

### Instrument

Results of the application of the first questionnaire on gastrointestinal symptoms in pwCF were published in 2017.^3^ The study was carried out with patients of all ages who attended the Jena University Hospital Cystic Fibrosis Centre in Jena, Germany. An updated version was subsequently applied in the same year, including CF patients from the same Reference Center, to measure the GI symptoms during the previous three months. Symptoms were grouped into four domains: abdominal pain, non-pain symptoms, subjective evaluation of the feces' frequency, form and color, and disorders of eating and appetite. A third publication presented the validation data for the questionnaire, which was renamed CFAbd-Score.^5^ The results showed that the questionnaire differentiated CF patients from healthy controls (17.3 ± 1.1 vs. 8.0 ± 0.7 points; p < 0.001; Cohen's *d* = 0.85). High item-domain loadings as well as good to excellent internal consistency and reproducibility. Cronbach's *α* = 0.70–0.92 and intra-class correlation coefficient = 0.932 indicated construct validity and reliability. The questionnaire has been adapted in the following countries: Germany, Austria, Switzerland, UK, Ireland, USA, Canada, Australia, Spain, Argentina, Portugal, Belgium, Netherlands, Denmark and Greece and has been validated in English, German, Greek and French.

The original questionnaire to be translated and cross-culturally adapted consisted of 28 questions grouped into five domains: pain (4 items), gastroesophageal reflux symptoms (3 items), disorders of bowel movement (8 items), disorders of appetite (5 items), and impact of these symptoms on QoL (8 items). Questions used a 6-point Likert scale, the Faces Pain Scale (0 to 10 for pain intensity), and two questions on the modified Bristol scale.^8^ The scores for each item ranged from never (0 points) to always (5 points) or cannot get worse (5 points). Items refer to a recall period of the preceding two weeks. The domain points were calculated as the sum of the items answered over the maximum item points per domain. Based on binary logistic regression coefficients, the domains were weighted, and the CFAbd-Score was calculated on a scale from 0 to 100 with higher values accounting for higher frequency and/or severity of symptoms.^5^

### Sample size

Currently, 180 patients are being followed up at the university CF-Reference Center for pediatric patients. A convenience sample including all patients over the age of six, was taken as eligible. Considering the inclusion and exclusion criteria described above, we interviewed 97 patients.

### Questionnaire application

As for pwCF, the questionnaire was administered after their routine visit at the outpatient clinic; their parents and/or legal guardians could assist them in case they were unsure about a question. Healthy individuals answered the questionnaire on their own, at school, with the help of one of the researchers whenever necessary, and after legal consent by their parents and/or legal guardians.

### Cross-cultural adaptation

The cross-cultural adaptation was based on the procedures suggested by Reichenheim and Moraes^9^ and included an assessment of conceptual and item equivalence, semantic assessment, operational, and measurement equivalence. For conceptual and item equivalence, the questionnaire was presented and discussed by the healthcare team.

Semantic equivalence was conducted by two independent professionals affluent German who initially made the translation from German into Brazilian spoken Portuguese. Their versions were then back-translated into German by two other physicians fluent in German and Portuguese. After that, the two forward and the two backward translations were compared and discussed by CF carers, pwCF, and their proxies, and a final version was obtained. Semantic assessment evaluated the denotative and connotative meanings of words. The final version was then tested on a group of 10 pwCF and/or parents/legal guardians to assess the comprehensibility.

The same group of professionals who worked on conceptual and item equivalence discussed operational equivalence. The discussion focused on the scale and order of presentation of the items used in the original questionnaire.

### Content validity

Eleven experts met to evaluate the content validity of the CFAbd-Score. The content validity index was calculated by dividing the number of experts who agreed with the adequacy of each item by the total number of experts. Four pediatric gastroenterologists, one psychologist, one dietitian, one physician specializing in nutrition, two pediatric pulmonologists, one microbiologist, and one social worker worked on this step. All the items were assessed as relevant by the committee of gastroenterologists. The coefficients obtained ranged from 0.8 to 1.0, indicating agreement between the experts on the relevance of questions. Nineteen items had a coefficient of 1, five 0.9, and two items 0.8, the average coefficient was 0.96.

### Validation

Excel worksheet editor and the Statistical Package for the Social Sciences were used to register and analyze data.

Total Cronbach's alpha coefficient was evaluated to measure the internal consistency of responses within a set of items of the instrument. Cut-off points considered were 0.70 ≤ Alpha < 0.80, indicating good internal consistency between items, and Alpha ≥ 0.80, considered high internal consistency.

Bartlett's test of Sphericity was used to test the null hypothesis that the correlation matrix is an identity matrix. The Kaiser-Meyer-Olkin Measure of Sampling Adequacy was used to verify the adequacy of the data for factor analysis.

Exploratory factor analysis with oblique rotation was conducted. By inspection of Scree plots and eigenvalues, the number of factors to be retained was identified. The internal consistency of the extracted domains was evaluated using Cronbach's alpha.

The Research Ethics Committee of the university approved the study protocol, under CAAE number: 42733520.9.0000.5404.

## Results

### Participants

From 180 patients seen at the Reference Center, 61 were under six years old, therefore not included, and of the remaining 119 patients, 14 reported lactose intolerance, not meeting the inclusion criteria, eight patients did not attend their appointments to be interviewed, finally were interviewed 97 pwCF (47 female), aged 6-38 years; median age 14.58 y (IQR: 9/19) and 105 healthy individuals (52 female), median age 15.10 y, (IQR: 9/20). Table 1 shows the sociodemographic characteristics of CF patients.

**Table 1:** Socio-demographic characterístics of pwCF interviewed in the validation study.

Variable	N	%
Sex		
Male	50	51.5
Female	47	48.5
Educational level		
Elementary	47	48.5
Secondary	30	31
Higher	05	15.5
Professional job	15	15.5
CFTR genotype		
allele 1		
F508DEL	77	79.3
Other	20	20.6
allele 2		
F508DEL	22	22.6
G542X	18	18.5
A561E	10	10.3
N1303K	3	3
Other	45	46.3
Age (years)		
6-11	36	37.1
≥ 12	31	31.9
≥ 18	30	30.9
CF abdominal manifestations		
Exocrine pancreatic insufficiency	78	80.4
History of meconium ileus	12	12.3
History of distal intestinal obstruction syndrome	7	7.2
History of rectal prolapse	2	2
CF-associated liver disease	22	22.6
CF-related diabetes	4	4.1

The internal consistency of the questionnaire, using all questions was 0.89 (Cronbach's alpha).

### Factor analysis

Bartlett's test, used to test the null hypothesis that the correlation matrix is an identity matrix, obtained p < 0.001.

**Figure 1:**
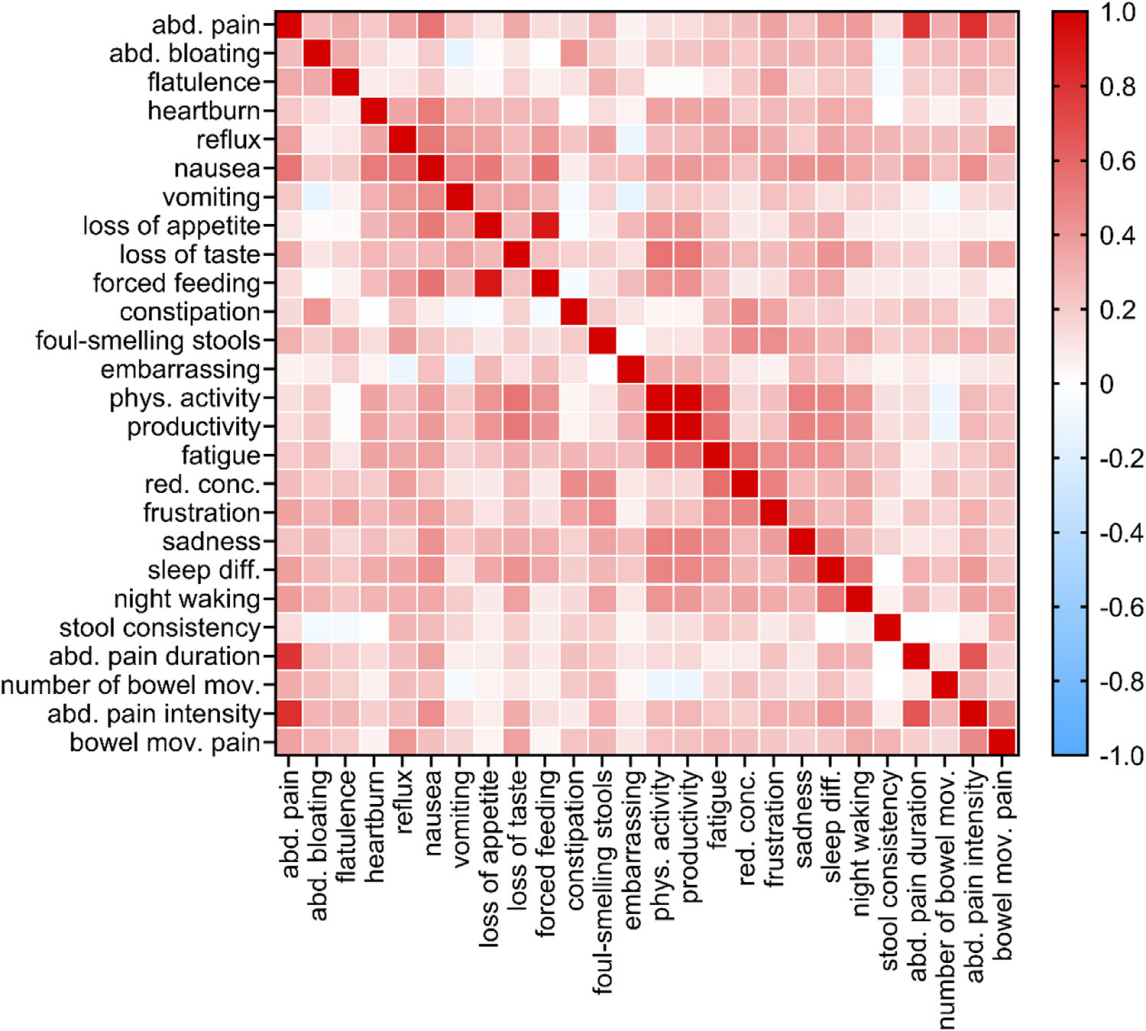
Correlation matrix plot showing the item-to-item correlation coefficients.

Item-to-item correlations: Figure 1 shows the correlation heatmap, a graphical representation of a correlation matrix representing the correlation between different variables. Besides the existing correlation between three pain items (pain prevalence, pain intensity, and pain duration), only the correlation coefficients corresponding to the bivariate correlations “reduced physical activity”- “reduced productivity” and “loss of appetite”- “force feeding” (0.997 and 0.906, respectively) were greater than 0.75.

**Figure 2:**
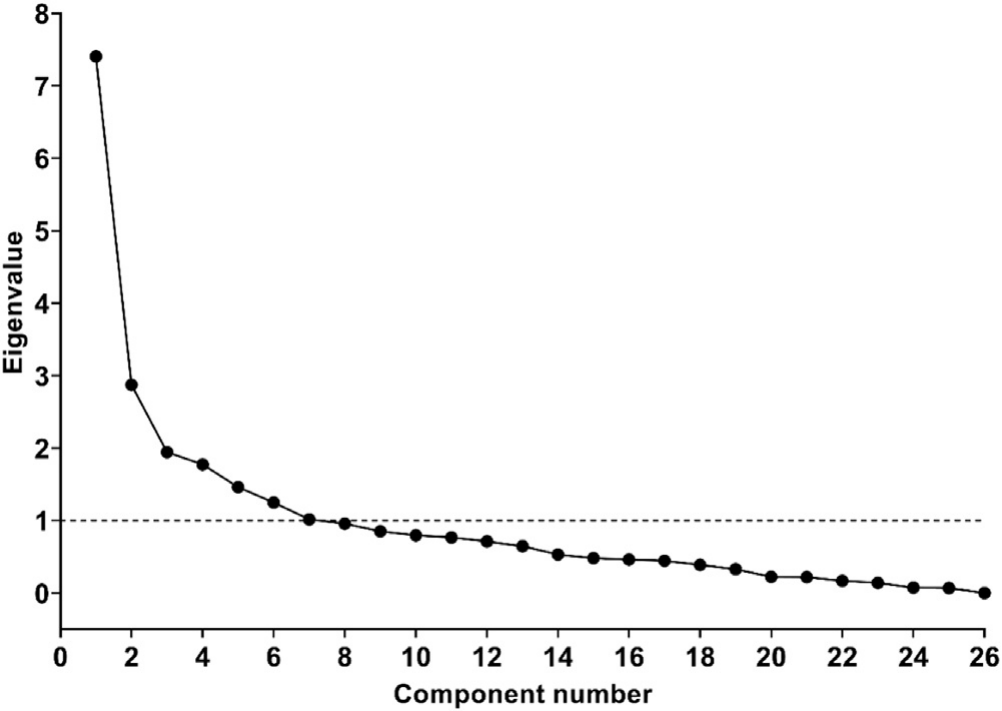
Scree plot showing the eigenvalues associated with the number of factors in the extraction order.

Figure 2 presents the Scree plot, a graphical representation of the eigenvalues associated with the number of factors in the extraction order. We followed the Kaiser criterion which suggests that we should only extract factors with eigenvalues greater than 1, resulting in seven retained factors, values shown in Supplementary Table.

The rotated component matrix resulting from including the seven factors with eigenvalues greater than 1 showed that one factor comprised only 2 items. A similar structure was observed when reducing the number of extracted factors to 6. The same structure was found with other methods of factor rotation, regardless of whether they assume orthogonality or not.

In contrast, a better and simpler factor structure resulted from restricting the number of factors extracted to 5 and selecting an orthogonal rotation (Varimax), which minimizes the number of variables having high loading on each factor. In all resulting structures, items related to embarrassment (“Sinto vergonha de problemas de digestão”) loaded on two factors. As one of these coefficients was negative, i.e. the greatest in absolute value (-0.471), it seemed therefore reasonable to include this item in Component 1 rather than in Component 5.

Internal consistency coefficients (Cronbach's alpha) were good to very good in the first four extracted factors (Table 2). The Cronbach's alpha for component 5, reflux, on the other hand, showed low internal consistency.

**Table 2:** Internal consistency coefficients (Cronbach's alpha) and percentage of variance explained for the first 5 factors shown in Supplementary Table.

	Variance explained (%)		
	Individual	Cumulative	Cronbach's alpha
Impairment of QoL	28.5	28.5	0.84
Pain symptoms	11.1	39.6	0.73
Disorders bowel movements	7.5	47.0	0.76
Disorders eating/appetite	6.8	53.9	0.81
Gastroesophageal reflux symptoms	5.6	59.5	0.54

## Discussion

The results obtained by this study showed that the CFAbd-Score, translated to spoken Portuguese and adapted to the Brazilian population, has a good level of internal consistency as an instrument to evaluate gastrointestinal symptoms in pwCF.

The tools available for QoL assessment in pwCF do not focus on gastrointestinal symptoms.^10^ The CFAbd-Score was specifically developed to assess the impact of gastrointestinal problems on the QoL.^11^^,^^12^ To date, there are no questionnaires in Portuguese available that assess the QoL in pwCF that include GI symptoms. This is the major contribution of the present study.

GI complications were found to markedly affect the general health, nutritional status, and QoL of pwCF.^1^ Tools that collect such data help evaluate QoL and aid in the diagnosis of clinical entities such as abdominal pain and constipation, with high-prevalence rates symptoms in pwCF.^13^^,^^14^ CFAbd-Score was developed in 2017[5] to be used to quantify the importance of GI symptoms in CF patients and as a tool for post-treatment evaluation. This use makes the validation of this instrument very timely since the Highly Effective Modulator Therapy has just been approved for being delivered by the Sistema Único de Saúde Brasileiro for the pwCF treated at Brazilian Reference Centers.^6^

Regarding the cross-cultural adaptation, the translation and back-translation of the questionnaire showed similarities with the original versions. The common main concept framed in each of the questions about GI symptoms may have enabled such similarity. The result not to be too complex, and terms do not appear to be critically influenced by the sociocultural context. The use of Greek and Latin roots, typical of many medical terms, allows concepts to be expressed in a few words that would otherwise require long phrases and sentences. The transfer of an idea conveyed by a medical term to the language spoken by the patient is commonplace in the medical setting. The translators, physicians and those familiar with the languages with which they work did not have any difficulties, and this might have made semantic equivalence easier, minimizing misinterpretations. The translation into Portuguese had excellent adequacy and was well accepted by the 10 patients who discussed the questionnaire during the content validity step. We attribute this performance to the simplified structure of the CFAbd-score, associated with questions more immersed in the impact of symptoms on QoL.

The terms included in the final version of the questionnaire were also selected based on the context where the questionnaire will be applied. Considering that the questionnaire would be administered to children, adolescents, and adults, the face-to-face application of the questionnaire was the most appropriate alternative. Considering that health professionals would apply the questionnaire, we choose using technical terms. And, also, during its implementation, the health professional could answer any questions the patients might have.

Concerning operational equivalence, some aspects require special considerations. The mode of application of a questionnaire that involves a broad age range, children from 6 years to patients above 20 years and up to 38, demands some operational customization. It is possible to get a more truthful answer when patients fill out a questionnaire rather than directly being asked by a physician or nutritionist. A self-administered questionnaire minimizes such a problem but may require proxies’ participation.

Content validity was assessed systematically through expert judgment, we obtained an excellent content validity index, by evaluating the instrument just with the team who work with pwCF in the Reference Center, including four pediatric gastroenterologists.

Bartlett's test is a statistical technique to test the null hypothesis that all the variables observed in a data set are uncorrelated with each other, i.e. that the population correlation matrix is an identity matrix. When applied to this sample, Bartlett's test indicated that there was statistical evidence to reject the null hypothesis which means variables are independent.

The KMO test plays an essential role in preparing for factor analysis, as it assesses whether the correlation matrix of the variables is robust enough to extract latent factors that explain the underlying structure of the data. The Kaiser-Meyer-Olkin Measures of Sampling Adequacy test was 0.702, indicating that the reduction of the strategic space through factor analysis would be adequate.

Two domains were accountable for the largest variation in the total score. These domains are related to pain and intestinal transit. The original questionnaire showed a high item-domain loading as well as good to excellent internal consistency and reproducibility, Cronbach's *α* range from 0.70 to 0.92. Domains and respective Cronbach's alpha are Impairment of QoL 0.92, Pain symptoms 0.85, Disorders of bowel movement 0.75, Disorders of eating/appetite 0.75, and gastroesophageal reflux symptoms 0.70. The present results were similar, with internal consistency coefficients (Cronbach's alpha) good in four extracted factors, abdominal pain: 0.84; abdominal bloating: 0.73; flatulence: 0.76; heartburn: 0.81 and low for reflux: 0.54. Some differences may be related to the differences in age groups comparing the cohort and the original one. Some issues related to gastro-esophageal reflux involve the age group of CF-patients. In the process of validity and reliability of the original instrument, the mean age of patients and healthy volunteers was 23.3 years and 25.5 years, respectively. The age range of the cohort was lower. It is known that reflux symptoms are more non-specific in children than in adolescents and that the prevalence of gastroesophageal reflux disease is higher in adult pwCF compared to children, additionally, adulthood is associated with increased esophageal acid exposure in CF-patients.^15^ Finally, adult patients tend to have more advanced lung disease, which may result in a greater respiratory disease burden compared to the relevance of GI symptoms.

In QoL studies, parents and guardians are proxies for their children since children may lack language and cognitive capabilities. The cohort of pwCF has included 36 individuals with over six and under 12 years old, 31 between 12 and 18 years old, and 30 over 18 years old. Probably, in the cohort, parents or guardians had to assess the QoL of their children on some items as “Sinto vergonha de problemas de digestão, Limitações em atividades cotidianas e no trabalho, Diminuição da produtividade laboral, Cansaço durante o dia, falta de concentração, Frustração/impaciência/irritabilidade e Tristeza”. Again, the difference in age range may explain differences in the internal consistency of recruited domains.

A limitation of this study is related to the sample size. We intended to include all patients seen in the Reference Center, but excluding those under six years old, losses, and those who referred lactose intolerance, we could include 97 patients. Although the sample is smaller than favorable, the cohort was sufficient when considering sample calculation based on the expected correlation, allowing analysis with significance. Other study limitations lay in the fact that we have not performed a test-retest reproducibility analysis. Agreement between the test and retest in the original CFAbd-Score allowed researchers to classify it as having good to excellent reproducibility, with an intra-class correlation coefficient = 0.932.

We concluded that the CFAbd-Score was translated and adapted according to all the proposed steps. Equivalences were adequately met, and the psychometric validity showed adequate performance.

## Funding source

This research did not receive any specific grant from funding agencies in the public, commercial, or not-for-profit sectors.

## Conflicts of interest

The authors declare no conflicts of interest.

## Appendix Supplementary materials

Image, application 1
