# Supplementary material for: Cross-cultural adaptation and validation of the CFAbd-Score for gastrointestinal symptoms in patients with cystic fibrosis
Source: J Pediatr (Rio J). 2024 Aug 10;101(1):82–8. doi: 10.1016/j.jped.2024.07.004 (PMC11763617; doi:10.1016/j.jped.2024.07.004)
Supplement: Supplementary file 1 [file mmc1.docx]

**JPED-D-24-00116 – Supplementary Material**

**Supplementary Table** Principal components and their corresponding percentage of variance explained.

|  |  | **Percentage of variance explained** | |
| --- | --- | --- | --- |
| **Component** | **Eigenvalue** | **Individual (%)** | **Cumulative (%)** |
| 1 | 7.408 | 28.493 | 28.493 |
| 2 | 2.876 | 11.063 | 39.556 |
| 3 | 1.944 | 7.477 | 47.033 |
| 4 | 1.775 | 6.826 | 53.859 |
| 5 | 1.462 | 5.622 | 59.481 |
| 6 | 1.248 | 4.799 | 64.281 |
| 7 | 1.016 | 3.908 | 68.188 |
| 8 | .956 | 3.677 | 71.866 |
| 9 | .852 | 3.278 | 75.143 |
| 10 | .797 | 3.067 | 78.211 |
| 11 | .766 | 2.944 | 81.155 |
| 12 | .713 | 2.741 | 83.896 |
| 13 | .648 | 2.492 | 86.388 |
| 14 | .533 | 2.051 | 88.439 |
| 15 | .483 | 1.859 | 90.297 |
| 16 | .463 | 1.782 | 92.079 |
| 17 | .447 | 1.719 | 93.798 |
| 18 | .389 | 1.497 | 95.295 |
| 19 | .329 | 1.267 | 96.562 |
| 20 | .224 | .862 | 97.424 |
| 21 | .219 | .842 | 98.266 |
| 22 | .168 | .646 | 98.911 |
| 23 | .139 | .533 | 99.445 |
| 24 | .075 | .075 | .289 |
| 25 | .068 | .068 | .261 |
| 26 | .001 | .001 | .006 |
